# M1-Type, but Not M4-Type, Melanopsin Ganglion Cells Are Physiologically Tuned to the Central Circadian Clock

**DOI:** 10.3389/fnins.2021.652996

**Published:** 2021-05-06

**Authors:** Adam R. Stinchcombe, Caiping Hu, Olivia J. Walch, Samuel D. Faught, Kwoon Y. Wong, Daniel B. Forger

**Affiliations:** ^1^Department of Mathematics, University of Toronto, Toronto, ON, Canada; ^2^Department of Ophthalmology and Visual Sciences, University of Michigan, Ann Arbor, MI, United States; ^3^Department of Neurology, University of Michigan, Ann Arbor, MI, United States; ^4^Department of Mathematics, University of Michigan, Ann Arbor, MI, United States; ^5^Department of Molecular, Cellular and Developmental Biology, University of Michigan, Ann Arbor, MI, United States; ^6^Department of Computational Medicine and Bioinformatics, University of Michigan, Ann Arbor, MI, United States; ^7^Michigan Institute for Data Science, University of Michigan, Ann Arbor, MI, United States

**Keywords:** electrophysiological modeling, circadian rhythm, photoentrainment, intrinsically photosensitive retinal ganglion cell, suprachiasmatic nuclei

## Abstract

Proper circadian photoentrainment is crucial for the survival of many organisms. In mammals, intrinsically photosensitive retinal ganglion cells (ipRGCs) can use the photopigment melanopsin to sense light independently from rod and cone photoreceptors and send this information to many brain nuclei such as the suprachiasmatic nucleus (SCN), the site of the central circadian pacemaker. Here, we measure ionic currents and develop mathematical models of the electrical activity of two types of ipRGCs: M1, which projects to the SCN, and M4, which does not. We illustrate how their ionic properties differ, mainly how ionic currents generate lower spike rates and depolarization block in M1 ipRGCs. Both M1 and M4 cells have large geometries and project to higher visual centers of the brain via the optic nerve. Using a partial differential equation model, we show how axons of M1 and M4 cells faithfully convey information from the soma to the synapse even when the signal at the soma is attenuated due to depolarization block. Finally, we consider an ionic model of circadian photoentrainment from ipRGCs synapsing on SCN neurons and show how the properties of M1 ipRGCs are tuned to create accurate transmission of visual signals from the retina to the central pacemaker, whereas M4 ipRGCs would not evoke nearly as efficient a postsynaptic response. This work shows how ipRGCs and SCN neurons' electrical activities are tuned to allow for accurate circadian photoentrainment.

## Introduction

Unlike rod and cone photoreceptors, which signal to the brain via second-and third-order retinal neurons, intrinsically photosensitive retinal ganglion cells (ipRGCs) communicate light information directly to the brain (Berson et al., 2002; Dacey et al., 2005; Hattar et al., 2006). Since their discovery in the early 2000s, ipRGCs have been investigated extensively, with multiple types and functional roles identified for the cells (Baver et al., 2008; Ecker et al., 2010; Li and Schmidt, 2018). M1-type ipRGCs project to the SCN, the master circadian pacemaker, and are essential for the photoentrainment of circadian rhythms. M1 cells have also been implicated in additional functions, including modulating light adaptation in the outer retina (Prigge et al., 2016) and mediating the pupillary light reflex (Güler et al., 2008). The other types, M2 through M6, appear to have similarly diverse roles in image- and non-image-forming vision (Sondereker et al., 2020). M4 cells have received attention for their role in image-forming vision and contrast sensitivity (Estevez et al., 2012; Schmidt et al., 2014; Zhao et al., 2014; Schroeder et al., 2018).

**Figure 1:**
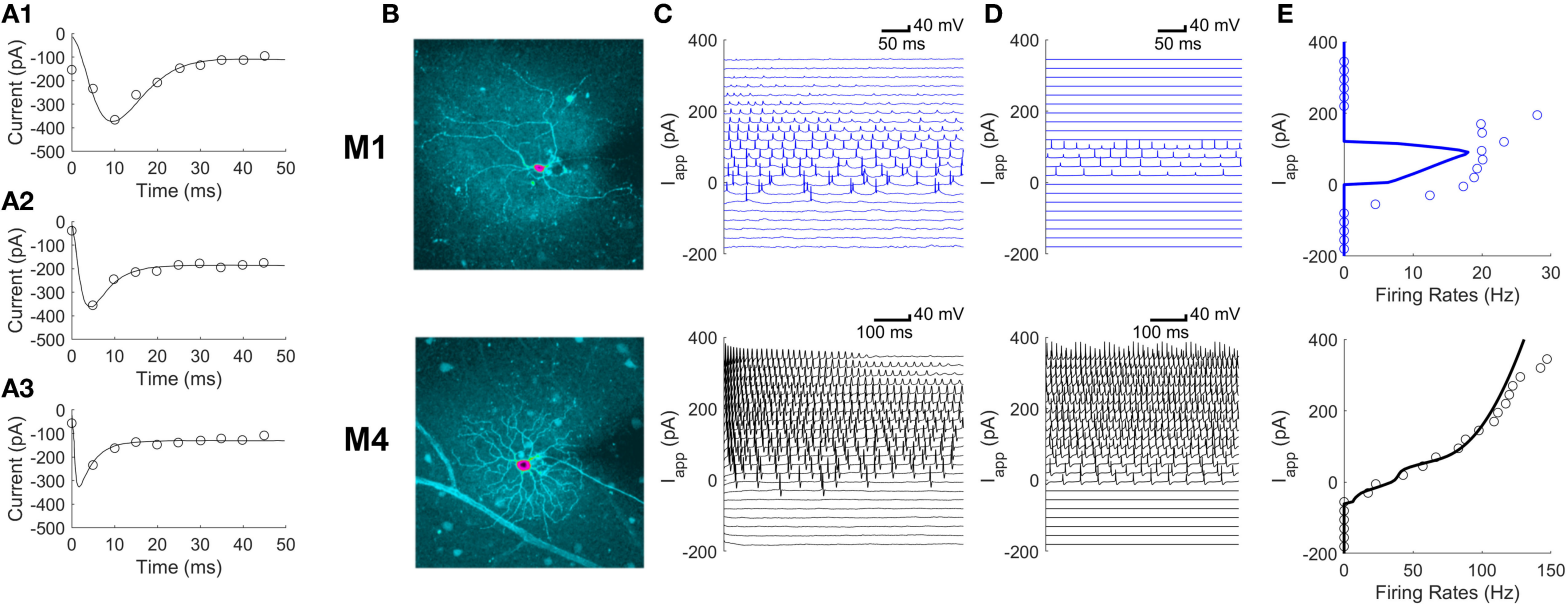
Differences in parameters of ionic currents can explain differences in firing patterns between M1 and M4 ipRGCs. **(A1–A3)** Voltage clamp Ca^2+^ currents averaged over 5 ms (circles) in a single M1 cell in response to depolarizing voltage steps of 30, 60, and 90 mV in amplitude for panels **(A1–A3)**, respectively. Fits of this kind were used to develop the model. **(B)** The intracellular dye fills of an M1 ipRGC (top) and an M4 ipRGC (bottom); pink centers mark the somas. **(C,D)** A model validation comparing experimental current-clamp data **(C)** to simulated data **(D)** in M1 cells (top) and M4 cells (bottom) at varying levels of the applied current, I_app_. **(E)** Comparing model (solid curve) and experimental (circles) firing rates in M1 cells (top) and M4 cells (bottom) at varying levels of I_app_. The firing rates were estimated from the current clamp responses in panels **(C)** and **(D)**.

As ipRGCs are spiking neurons, Hodgkin-Huxley style models can be used to approximate their firing behavior and electrophysiology. The specific electrophysiological properties of ipRGCs are strongly type-dependent. The two types this paper focuses on, M1 and M4, have markedly different morphologies and physiologic properties (Figure 1). In mouse M1 cells, the threshold for spiking is around −57 mV, while for M4 cells, the threshold is around −73.5 mV (Hu et al., 2013). Similarly, M1 cells fire with a maximum average rate of around 25 Hz, while M4 cells can fire at more than twice that rate (Hu et al., 2013).

Many ionic currents have been identified in mouse ipRGCs (Hu et al., 2013). Other electrophysiological characteristics, such as input resistances and firing rates, have been quantified and shown to vary significantly among ipRGC types (Schmidt and Kofuji, 2009, 2011; Hu et al., 2013). The diversity of electrical properties, both between and within ipRGC types, alongside the cells' distinct morphologies suggests that no single model implementation can capture all ipRGC behavior. Indeed, past work modeling the firing rate properties of these cells (Walch et al., 2015) found that ipRGC type-specific parameters were necessary to capture the varying behaviors observed across types. Despite experimental data quantifying these cells' ion channel dynamics, ipRGC-specific mathematical models have not yet been built, which capture these dynamics.

Since M1 and M4 are ipRGC types with significantly different firing properties, they present two definitive case studies for modeling the diversity of ipRGCs. In particular, M1 cells fire slowly and enter into depolarization block, whereas M4 fire at much faster rates (Hu et al., 2013). Here, we develop two Hodgkin-Huxley style models for M1 and M4 ipRGCs and use those models to simulate possible ways by which they could signal to the SCN. Stinchcombe and colleagues' mathematical model (Stinchcombe et al., 2017) considered a simple (non-ionic) model of ipRGC electrical activity but was able to identify properties of ipRGC-to-SCN network connectivity, on which we base our simulations.

### Past Modeling of Retinal Ganglion Cells

Conventional, non-melanopsin-expressing retinal ganglion cells (RGCs) in the tiger salamander have been modeled extensively by Fohlmeister and Miller (Fohlmeister et al., 1990; Fohlmeister and Miller, 1997a,b). Their model, in addition to containing Na^+^, K^+^, and an A-type current includes voltage-gated Ca^2+^ and Ca^2+^-activated K^+^ currents that depend explicitly on internal Ca^2+^ concentration. Intriguingly, Fohlmeister and Miller (1997b) demonstrated that incorporating morphology into their simulations could account for the large range of firing rates observed in different types of RGCs. Models have also explained variation in RGC responses due to inherent differences, e.g., ON and OFF RGCs (Kameneva et al., 2011), as well as intrinsic and extrinsic noise (Shi et al., 2019; Sekhar et al., 2020).

While M1 ipRGCs are nominally like non-photoreceptive RGCs, their electrophysiology may be more similar to their postsynaptic cells in the SCN than to other RGCs. In particular, SCN neurons and M1 ipRGCs have both been shown to fire at relatively slow rates and enter depolarization block in the presence of sufficiently high applied current (Belle et al., 2009; Diekman et al., 2013; Hu et al., 2013; Milner and Do, 2017), while conventional RGCs tend to fire at increasingly faster rates with increasing current injection (O'Brien et al., 2002; Wong et al., 2012). Thus, we wondered if these two cell types were tuned to reliably transmit information, for example as measured by Irwin and Allen (Irwin and Allen, 2007) who see consistent stimulation of the retinohypothalamic tract (RHT) leading to action potentials in SCN neurons. Meng et al. (2018) found that large external current stimulation to conventional RGCs can prevent somatic spiking but nonetheless conduct spikes in their axons. We therefore test the hypothesis that ipRGCs in depolarization block can have spiking axons.

## Materials and Methods

### Experimental Methods

All animal procedures were approved by the Institutional Animal Care and Use Committee at the University of Michigan. The mathematical model was based on voltage-clamp recordings of ionic currents and current-clamp recordings of spiking activity, obtained from green fluorescent protein-labeled ipRGCs in flat-mount mouse retinas (Ecker et al., 2010) in the whole-cell configuration, as described previously (Hu et al., 2013). Intracellular dye fills enabled the identification of the recorded ipRGCs based on morphological criteria: M1 cells had medium-sized somas and sparse dendrites stratifying in the OFF sublamina, whereas M4 cells had giant somas and dense, radiate dendrites stratifying near the retinal surface (Ecker et al., 2010; Estevez et al., 2012).

Two kinds of intracellular solutions were used. For all current-clamp recordings and voltage-clamp recordings of K^+^ currents, we used a K^+^-based solution containing (in mM) 120 K-gluconate; 5 NaCl; 4 KCl; 10 HEPES; 2 EGTA; 4 Mg-ATP; 0.3 Na-GTP; 7 Tris-phosphocreatine; 0.1% Lucifer Yellow; and KOH to adjust pH to 7.3. For voltage-clamp recordings of Ca^2+^ and Na^+^ currents, we used a Cs^+^-based solution to reduce K^+^ currents, which contained (in mM) 120 Cs-methanesulfonate; 5 NaCl; 4 tetraethylammonium chloride; 10 HEPES; 2 EGTA; 4 Mg-ATP; 0.3 Na-GTP; 7 Tris-phosphocreatine; 0.1% Lucifer Yellow; and CsOH to adjust pH to 7.3. The liquid junction potential was computed with CLAMPEX software, which was found to be around 13 mV for the K^+^-based solution and around 10 mV for the Cs^+^-based solution.

Five kinds of bathing solutions were used. To record voltage-gated Ca^2+^ currents, the holding potential was −80 mV, and a series of voltage steps was first applied in the presence of a 5 mM Ca^2+^ Ringer containing (in mM) 105.4 NaCl; 20 tetraethylammonium chloride; 10 CsCl; 5 CaCl_2_; 1.24 MgCl_2_; 10 HEPES; 16 D-glucose; 0.5 L-glutamine; 0.0003 tetrodotoxin to block voltage-gated Na^+^ channels; and NaOH to adjust pH to 7.4. The same voltage steps were then presented again in the presence of a 6.24 mM Mg^2+^ Ringer which was identical to the 5 mM Ca^2+^ Ringer except that 5 mM CaCl_2_ had been replaced by equimolar MgCl_2_ to block Ca^2+^ channels. These two sets of responses were subtracted to isolate voltage-gated Ca^2+^ currents.

To record K^+^ currents, including both voltage-gated and Ca^2+^-activated K^+^ currents, we used a holding potential of −90 mV and applied a series of depolarizing voltage steps in the presence of a Ringer containing (in mM) 120 NaCl; 3.6 KCl; 1.15 CaCl_2_; 1.24 MgCl_2_; 22.6 NaHCO_3_; 16 D-glucose; 0.5 L-glutamine; and 0.0003 tetrodotoxin. This solution was gassed continuously with 95% oxygen 5% carbon dioxide. With only 1.15 mM Ca^2+^ in the bath, the amplitudes of voltage-gated Ca^2+^ currents were fairly negligible. After performing leak subtraction offline using Clampfit software (Molecular Devices, San Jose, CA), all remaining current responses were assumed to be K^+^ currents.

To record voltage-gated Na^+^ currents, the bathing solution was a K^+^-blocking Ringer containing (mM) 56.0 NaCl; 57.6 tetraethylammonium chloride; 10 CsCl; 1.24 MgCl_2_; 1.15 CaCl_2_; 22.6 NaHCO_3_; 16 D-glucose; and 0.5 L-glutamine. This solution was gassed with 95% oxygen 5% carbon dioxide. Voltage steps were first applied with a holding potential of −80 mV to permit voltage-gated Na^+^ channel activation, and then again with a holding potential of −20 mV to inactivate these channels, and the two sets of responses were subtracted to isolate voltage-gated Na^+^ currents. To improve space-clamp quality, we used a micromanipulator to move a glass micropipette around the soma to sever some of the dendrites, before obtaining whole-cell recording from the soma.

To record spiking responses to current steps, the bathing solution was artificial cerebrospinal fluid containing (in mM) 120 NaCl; 3.6 KCl; 1.15 CaCl_2_; 1.24 MgCl_2_; 22.6 NaHCO_3_; 16 D-glucose; and 0.5 L-glutamine. This solution was gassed with 95% oxygen 5% carbon dioxide. A negative holding current was applied to hyperpolarize the cell to around −80 mV, and a series of depolarizing current steps was presented to induce action potentials.

### Mathematical Methods

#### A Model of the Soma

Conductance-based models (also known as Hodgkin-Huxley style models) with voltage-gated sodium, potassium, and calcium channels and a chloride leak channel were fit using voltage-clamp data and MATLAB's fminsearch function. Specifically, the model parameters were selected to minimize the sum of square differences in current, averaged over many voltage-clamp experiments: Ca^2+^ currents in 21 M1 cells and eight M4 cells (Hu et al., 2013); K^+^ currents in 22 M1 cells and 15 M4 cells (Hu et al., 2013); and Na^+^ currents in one M1 cell and one M4 cell with most dendrites removed. Depolarizing voltage steps of 30, 60, and 90 mV in amplitude were used and the measured currents were averaged over intervals of 5 ms before comparison with the model. The models were validated using current-clamp data in 17 M1 and 13 M4 cells. The model equations are presented in the Results section. MATLAB code for simulating the models is available on ModelDB (McDougal et al., 2017) at http://modeldb.yale.edu/267026.

#### A Model of the Axon

A spatial Hodgkin-Huxley model (Keener and Sneyd, 1998) is used to capture the qualitative aspects of the propagation and signal attenuation down the RHT. In particular, the voltage *V*(*x, t*) at position *x* along the axon is governed by

$$\begin{array}{l} C\frac{\partial V}{\partial t}=D\frac{\partial^{2}V}{\partial x^{2}}+I_{Na}+I_{K}+I_{Cl}, \end{array}$$

in which *C* is the axonal membrane capacitance; *D* is the voltage diffusivity; and *I*_*Na*_, *I*_*K*_, *I*_*Cl*_ are the sodium, potassium, and chloride currents, respectively. These currents make the axon excitable (Keener and Sneyd, 1998) allowing it to transmit its input from an ipRGC. The value of *D* is selected to match the RHT propagation delay of 50 ms observed experimentally (Wong et al., 2007; Mouland et al., 2017), less the synaptic delay of 4.7 ms (Moldavan and Allen, 2010). The experimentally measured ipRGC membrane potentials *V*_*data*_ are input to the axon model via a Robin boundary condition at the somatic end of the axon. In particular, $\frac{\partial V}{\partial x}\left(0,t\right)\;=\kappa \;(V\left(0,t\right)-V_{data}$), as though the soma is attached to the axon with a conductance κ (Abbott and Dayan, 2001). A no-flux (i.e., no axial current) boundary condition is used at the axon terminal, i.e., $\frac{\partial V}{\partial x}\left(L,t\right)=0$. At the axon terminal at *x* = *L*, the *filtered* output is recorded. The partial differential equation is solved numerically with a custom-written finite difference scheme in MATLAB.

#### A Model of the RHT-SCN Synapse

The model for the synaptic connections from ipRGCs to the SCN is described in Stinchcombe et al. (2017). Specifically, AMPA and NMDA glutamate synapses result in an additional SCN current of

$$\begin{array}{l} I_{RHT}=-g_{AMPA}s_{AMPA}\left(V_{SCN}-E_{AMPA}\right)\\ \;-g_{NMDA}s_{NMDA}B\left(V_{SCN}-E_{NMDA}\right), \end{array}$$

in which *B* = 1/(1 + exp (− (*V*_*SCN*_ − *MgVT*)/16.13)). The synaptic gating variables *s*_*AMPA*_, *s*_*NMDA*_ are driven open by M1/M4 ipRGC spikes:

$$\begin{array}{l} \frac{ds}{dt}=a_{r}\frac{T_{max}}{1+exp\left(-\frac{V_{ipRGC}-V_{T}}{K_{p}}\right)}\left(1-s\right)-a_{d}s, \end{array}$$

in which *s, a*_*r*_, *a*_*d*_, *T*_*max*_, *V*_*T*_, *K*_*p*_ are different for the AMPA and NMDA synapses. Parameter values and an explanation of this synaptic model is given in Stinchcombe et al. (2017). The electrical activity of SCN neurons is modeled by a Hodgkin-Huxley style model that is time-of-day dependent through regulation of the Ca^2+^-gated K^+^ and K^+^ leak conductances (Sim and Forger, 2007; Diekman et al., 2013; DeWoskin, 2015). The RHT-SCN synapse model has been validated against experimental observations from Irwin and Allen (2007) and Moldavan and Allen (2010).

## Results

### Markedly Different Parameters Are Needed to Capture the Different Behaviors in M1 and M4 Cells

The parameters of a conductance-based model were fit to voltage-clamp experiments as described in the section “A Model of the Soma” separately for M1 and M4 ipRGCs. Representative calcium current fits for an M1 ipRGC are shown in Figures 1A1–A3 for three step voltages. The conductance-based model is described by differential equations for the membrane voltage dynamics,

$$\begin{array}{l} C_{m}\frac{dV}{dt}=-g_{Na}m^{3}h\left(V-E_{Na}\right)-g_{K}n^{4}\left(V-E_{K}\right)\\ \;-g_{Ca}rf\left(V-E_{Ca}\right)-g_{L}\left(V-E_{L}\right)+I_{app}, \end{array}$$

and for the gating variable dynamics,

$$\begin{array}{l} \frac{ds}{dt}=\frac{s_{\infty }\left(V\right)-s}{\tau_{s}\left(V\right)}. \end{array}$$

The variable *s* stands-in for any of the gating variables *m*, *h*, *n*, *r*, or *f*. The fit parameter values differ significantly between M1 and M4 ipRGCs. In particular, the M1 ipRGCs have voltage-dependent equilibrium gating variable values and timescales given by

$$\begin{array}{l} m_{\infty }=\frac{1}{1+\exp\left(-0.25V-4.5\right)},\\ h_{\infty }=\frac{1}{1+\exp\left(0.36V+11.2\right)},\\ r_{\infty }=\frac{1}{1+\exp\left(-0.27V-3.33\right)},\\ f_{\infty }=\frac{1}{1+\exp\left(0.031V+4\right)},\\ n_{\infty }=\frac{1}{\sqrt[{4}]{1+\exp\left(-0.117V+0.823\right)}},\\ \tau_{m}=\exp\left(-0.013V-3.2\right),\\ \tau_{h}=0.12+\exp\left(-0.28V-5.18\right),\\ \tau_{r}=0.738,\\ \tau_{f}=\exp\left(-0.0091V+0.582\right),\\ \tau_{n}=\exp\left(-0.0294V-0.45\right). \end{array}$$

The same quantities for M4 ipRGCs are

$$\begin{array}{l} m_{\infty }=\frac{1}{1+\exp\left(-0.124V-3.4\right)},\\ h_{\infty }=\frac{1}{1+\exp\left(0.178V+9.75\right)},\\ r_{\infty }=\frac{1}{1+\exp\left(-0.130V-2.2\right)},\\ f_{\infty }=\frac{1}{1+\exp\left(0.015V+3.87\right)},\\ n_{\infty }=\frac{1}{\sqrt[{4}]{1+\exp\left(-0.0575V+1.317\right)}},\\ \tau_{m}=\exp\left(-0.0061V-4.49\right),\\ \tau_{h}=0.0324+\exp\left(-0.138V-5.318\right),\\ \tau_{r}=0.1973,\\ \tau_{f}=\exp\left(-0.0044V-0.603\right),\\ \tau_{n}=\exp\left(-0.0144V-0.729\right). \end{array}$$

These parameter sets correspond to local minima of the least-squares fitting procedure. Although the parameter space was explored extensively, there may be similar or better fits to the voltage step data.

Although M1 and M4 cells have different morphologies (Figure 1B), we found that the difference in their firing patterns could be explained by differences in the parameters of their ionic currents. Figure 1C shows voltage traces for M1 and M4 cells in response to different applied currents. Figure 1D shows the model's predictions of these voltage traces and the corresponding firing rates are quantified in Figure 1E.

**Figure 2:**
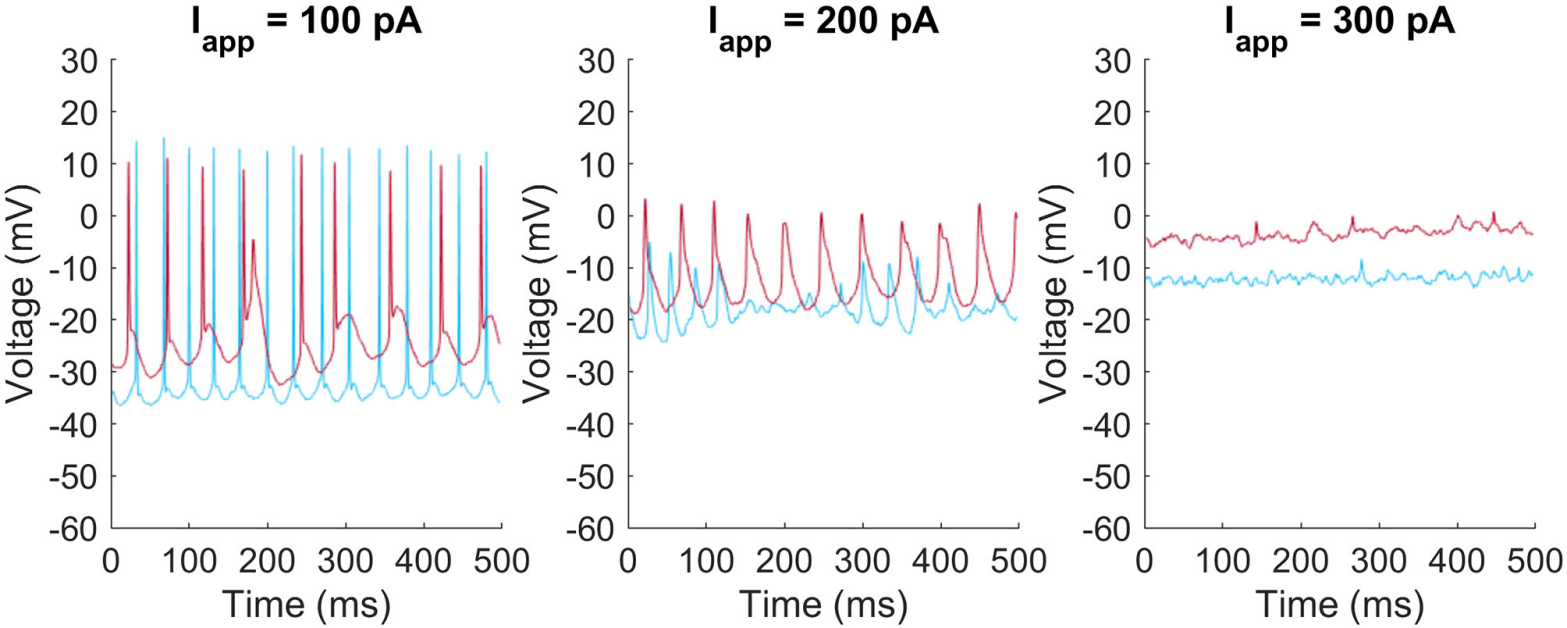
Heterogeneity of firing patters in M1 ipRGCs. Two M1 cells (red and blue) respond at different rates and with different resting membrane potentials to injected currents of *I*_app_ = 100 pA **(left)**, 200 pA **(middle)**, and 300 pA **(right)**.

The M4 model faithfully reproduces the faster firing rates seen in M4 ipRGCs. The M1 model reproduces the slower firing pattern seen in M1 cells as well as the depolarization block seen when neurons have larger excitatory applied currents. We also found that there was more diversity in the firing behavior of M1 cells than the cell shown in Figure 1C. Examples of differences between individual M1 cells are shown in Figure 2. These included differences in the rest membrane potential, firing rate, or when neurons start or stop firing. Electrophysiological recordings from all ganglion cell types show significant variation, due to varying degrees of cell health, different pressures exerted by the electrode onto the soma, different somatic locations of the electrode, variation in series resistance, the electrode inadvertently damaging one or more dendrites during its approach to the soma, varying levels of light adaptation, etc. Thus, it remains unclear whether the variations previously observed by Milner and Do (2017) and Hu et al. (2013) within each ipRGC type are truly biologically relevant, or merely experimental artifacts. We verified that <10% perturbations of the M1 ipRGC model parameters were able to reproduce the firing rate variability observed in Hu et al. (2013) while keeping the same basic properties of slow firing or depolarization block.

### Mathematical Modeling Can Reproduce Signaling at the End of the Axon

**Figure 3:**
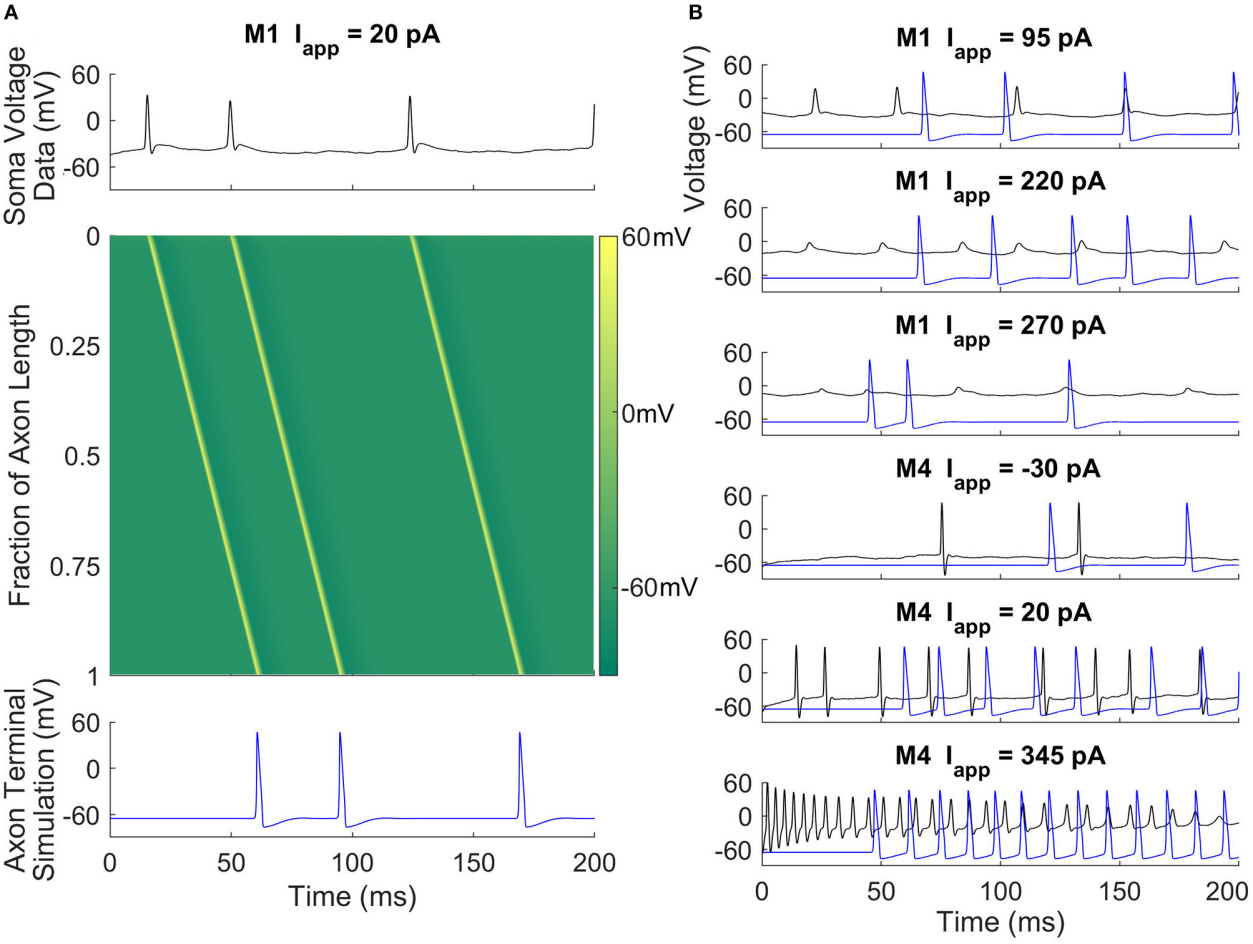
Axonal action potential propagation in M1 and M4 ipRGCs. **(A)** A space-time colored contour plot (middle) showing the propagation of action potentials in the RHT given input taken from a current-clamp recording of an M1 cell (top) and the simulated output at the axon terminal (bottom). There is an approximate 45 ms propagation delay along the axon. **(B)** Recorded soma voltage (black) and predicted voltage near the axon terminal (blue) for M1 and M4 cells at three different current-clamp values. The simulation suggests that M1 cells can signal to the SCN despite experiencing depolarization block or attenuated action potentials in the soma.

Models can be used to answer questions about M1 and M4 cells' roles and functions. ipRGCs have long axons that project to the hypothalamus and other brain regions. Given this, one can ask how the signal at the soma changes after propagation down a long axon. To address this, we simulate the RHT connecting the ipRGC somas to the SCN using a model of the axon (Figure 3). Data from the current-clamp experiments are used to prescribe soma voltage, and we use the spatial Hodgkin-Huxley model to predict how those somatic voltages are transmitted to the axon terminal. Both M1 and M4 were able to faithfully send signals down the axon. We find that even in depolarization block states (i.e., no large spikes) for M1, our mathematical model agrees with the experimental measurements of voltage further down the axon (Milner and Do, 2017). Interestingly, when the neuron is near depolarization block and signals are irregular, they nonetheless are translated to uniform signals in the axon. This also agrees with the data from Milner and Do (2017). Having seen how somatic signals propagate along the axon, we next looked at a model of the synapse with SCN neurons.

### M1 ipRGCs Are Tuned to the SCN

**Figure 4:**
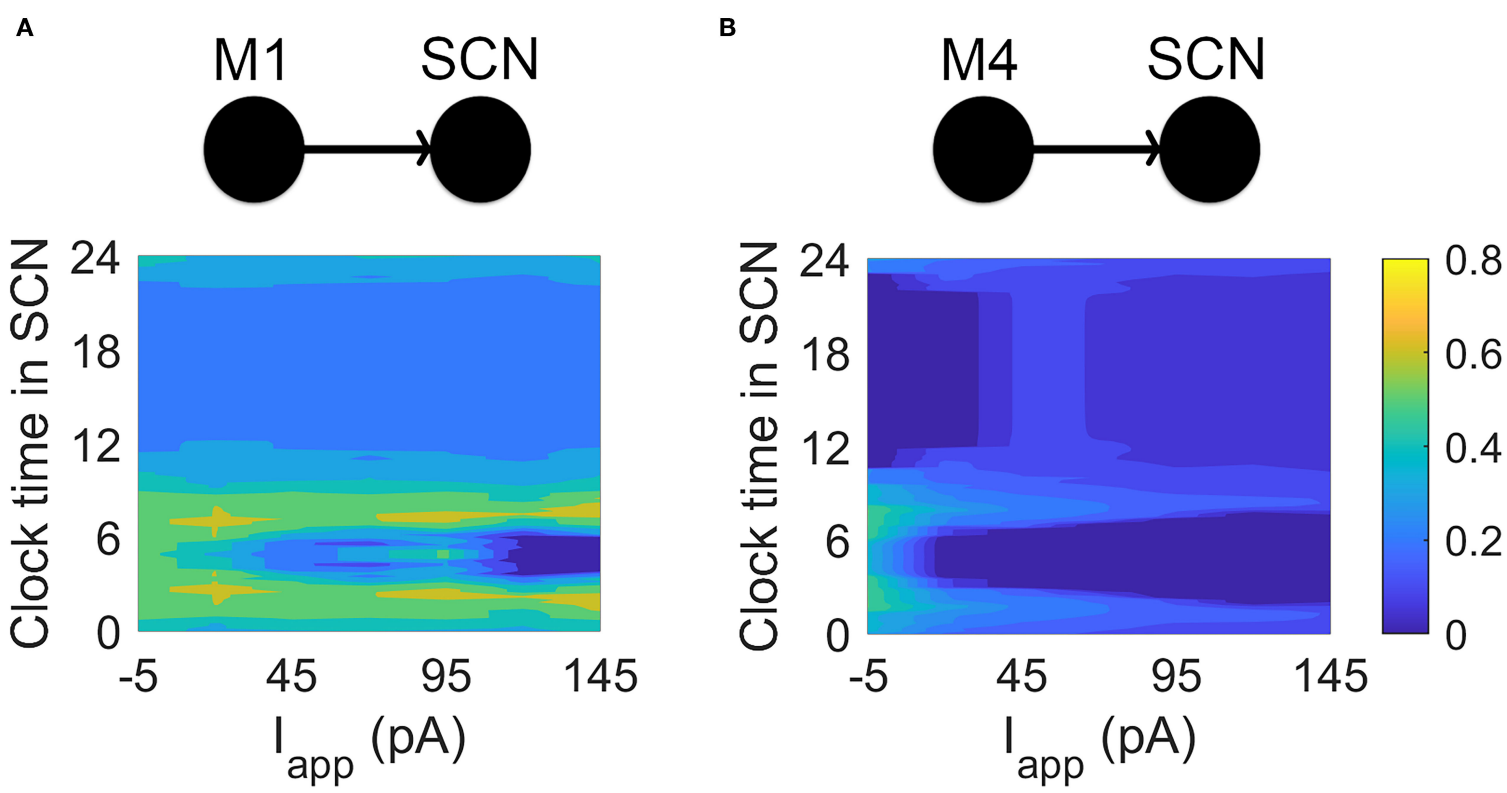
Simulating connections between M1, M4, and SCN cells. **(A)** Current-clamp data from M1 cells is passed via model glutamate synapses to a simulated SCN cell. While SCN neurons are able to match the firing rates of M1 cells, they are unable to match the firing rates of M4 cells. The heat maps show the ratio (output SCN firing rate)/(input ipRGC firing rate) at different times of the circadian day and for different applied currents. For the M1-SCN connection, several input currents and clock times are able to achieve near 1:1 input firing rate:output firing rate (yellow in the heat map). **(B)** Current-clamp data from M4 cells is passed via a simulated— and non-physiological—glutamate synaptic model to a simulated SCN cell. SCN cells are unable to match the firing rates of the M4 cells, as seen in the heat map, which again shows the ratio (output SCN firing rate)/(input ipRGC firing rate) at different circadian times and for different applied currents. For the M4-SCN connection, the SCN at best captures one fifth of the action potentials sent by the M4 cell.

A natural question is whether the differences between M1 and M4 electrophysiology are related to their functions in non-image and image-forming vision, respectively. To address this, we connected current-clamp data from M1 and M4 ipRGCs to simulated SCN cells with a physiologically realistic model synapse. We simulated projections from M1 and M4 cells to a SCN neuron in different electrical states representing their electrical activity at different times of the day. While M1 cells reach a relatively low peak firing rate before entering depolarization block, M4 cells can fire at much higher rates. When M1 data is passed to a simulated SCN neuron, the SCN is able to match its firing rate (Figure 4). When the same is done for an M4 cell (a connection that does not occur in nature), the simulated SCN cannot fire quickly enough to match the firing rate, even at all clock times. It has been directly observed by Irwin and Allen (2007) that RHT activation faithfully causes action potentials in SCN neurons. We assume a one-to-one relationship between ipRGC firing and SCN firing, in agreement with Irwin and Allen (2007). However, more complex firing may also emerge, for example having SCN neurons entrained at sub (half) or super (twice) harmonic firing rates. Future experimental studies should be aware of these possibilities. Energetically, this means that M4 cells would be less efficient than M1 cells at projecting to the SCN, as their higher firing rates could not translate to SCN neurons.

## Discussion

M1 and M4 cells highlight the extreme diversity present among ipRGCs. This diversity is reflected in their distinct electrophysiological properties and in the very different parameters needed to fit their firing patterns. We developed mathematical models of M1 and M4 ipRGC electrophysiology using the Hodgkin-Huxley formalism. We used these models to demonstrate how their distinct ion channel dynamics can reproduce the firing rates seen in ipRGCs. Nearly all parameters differed between M1 and M4 ipRGCs showing that not just one current accounts for the differences between the cells. Consistent with their slower firing rates, the time constants are much longer for M1 ipRGCs than M4 ipRGCs (e.g., the time constant for the Ca^2+^ gating variable τ_*r*_ is three times larger for M1 ipRGCs). The half-saturation values of the gating variable steady states are smaller for M1 ipRGCs than M4 ipRGCs which suggests a mechanism for their late firing. Also, the transition slopes of the gating variable steady states are larger for M1 ipRGCs accounting for their propensity to enter depolarization block.

We were interested in more than reproducing observed firing behaviors in these cells. By simulating an M1 ipRGC in depolarization block in a model of the RHT, we were able to demonstrate M1 signaling is faithfully reproduced at the end of the axon. In addition, we sought to understand ways in which M1 and SCN cells were, or were not, tuned to work together. To explore this, we coupled current-clamp recordings of both M1 and M4 ipRGCs to the SCN via a simulated RHT. Through this coupling, we were able to demonstrate how M1 projections to SCN cells are energetically efficient, as they fire only at rates capable of translating to the SCN cells. Firing of the kind observed in M4 cells, in contrast, is too fast to be converted effectively by SCN cells. In this way, the electrophysiological “form” of M1 cells is tuned to their function.

Our work also opens up future work to explore the roles of ipRGCs in subconscious vision. Stinchcombe et al. (2017) argue that action potentials in the SCN may carry spatial visual information, whereas depolarization block may synchronize circadian rhythms in the SCN. Further work could explore the different signaling modes between ipRGCs and SCN neurons in response to other visual signals. Our work also opens up the possibility that the electrical activity in the soma of ipRGC is different from that at the axon's end. Whether the signal just attenuates along the axon or fundamentally changes (e.g., changing from depolarization block to repetitive firing) remains to be experimentally verified, keeping in mind that this change may only be seen at the farthest distances from the retina. Our mathematical models of M1 and M4 ipRGCs could be useful in additional studies parsing out rod and cone inputs with the photoreception that occurs within ipRGCs. Morphological modeling may be needed to address these questions fully.

## Data Availability Statement

The original contributions presented in the study are included in the article/supplementary material, further inquiries can be directed to the corresponding author.

## Ethics Statement

The animal study was reviewed and approved by The University of Michigan Institutional Animal Care and Use Committee.

## Author Contributions

CH collected data. KW, AS, OW, SF, and DF analyzed data. AS, OW, SF, and DF developed mathematical models. AS, OW, KW, and DF wrote the manuscript. DF and KW obtained funding and oversaw the project. All authors contributed to the article and approved the submitted version.

## Conflict of Interest

OW is the CEO and DF the CSO of Arcascope, a company that makes software for circadian rhythms. The University of Michigan is a part owner of Arcascope. Arcascope did not sponsor this research. The remaining authors declare that the research was conducted in the absence of any commercial or financial relationships that could be construed as a potential conflict of interest.
